# The Road Ahead and Challenges of Revenue Cycle Management in Saudi Governmental Hospitals

**DOI:** 10.3390/healthcare11202716

**Published:** 2023-10-12

**Authors:** Zainab Alradhi, Abdullah Alanazi

**Affiliations:** 1 Health Informatics Department, King Saud Ibn Abdulaziz University for Health Sciences, Riyadh 11481, Saudi Arabia; 2 King Abdullah International Medical Research Center, Riyadh 14611, Saudi Arabia

**Keywords:** revenue cycle management (RCM), governmental hospitals, financial sustainability, health financial

## Abstract

Healthcare providers use revenue cycle management (RCM) to track patient billing and revenue. The revenue cycle collects data from various systems and compiles it into a single RCM system connected to payers. Effective system integration improves revenue and financial stability. The aim is to assess RCM feasibility in Saudi Arabia’s governmental hospitals, examine financial management, and recommend practical implementation. In this study, healthcare leaders were interviewed face-to-face and via audio recording to collect qualitative data in response to semi-structured questions. Key informants from seven main hospitals were interviewed. Respondents understood RCM and identified internal and external challenges in hospital financial management. Government hospitals face accountability obstacles. Two of the seven surveyed hospitals operate business clinics using a fee-for-service model. The billing system is not integrated with the information system. The RCM system faces challenges such as unclear vision, lack of accountability, staff resistance, process redesign, and importance of project management. Despite these challenges, respondents still value RCM and recognize its importance in improving hospital revenue management. Effective implementation of RCM requires significant transformational processes, including vision, governance, accountability, proper training, and effective monitoring and evaluation processes. Communication should also be emphasized, and the patient’s perspective must be brought into focus. Involving all stakeholders can create direct and holistic patient benefits; therefore, bringing them on board is crucial. New approaches are required to enhance healthcare in Saudi Arabia, addressing gaps in revenue optimization and RCM. Future research should evaluate the move from government-funded to self-operated hospitals, providing a better understanding of the challenges and opportunities.

## 1. Introduction

Effective revenue cycle management is essential for healthcare organizations; for example, in the United States, approximately 25% of nonprofit hospitals are experiencing negative margins. Healthcare providers must optimize their revenue cycle management (RCM) processes to maintain financial stability and provide quality patient care [1]. A study examined how revenue cycle management affects hospital profitability and equity capital. Two critical financial indicators were identified: patient revenue and revenue collection speed. These factors influence overall financial sustainability [2].

Revenue cycle management (RCM) is the system in the hospital that manages billable events throughout a single healthcare process and generates revenue as a result. Therefore, RCM tracks and manages patient billing, from scheduling an appointment, checking coverage eligibility, and receiving the care to receiving the final payment [3]. Thus, information is collected from different clinical and administrative systems, including but not limited to, Admission, Discharge, and Transfer (ADT) system, scheduling, bed management, health information management systems, and departmental and ancillary systems. Hence, clinical coders are generated based on diseases and clinical interventions, and claims are generated, scrubbed, and submitted to third-party payers. Payment and reimbursements are regulated by a national regulatory body, in addition to specific regulations imposed by third-party payers. This process is complicated due to varying payments for a single service based on provider, location, reimbursement, and regulations [4]. Traditionally, processes of RCM revolve around back-end tasks like billing and collecting payments. However, a greater focus on front-end tasks has emerged with recent technological advances like online scheduling, a platform with third-party payers, and hospital information systems, i.e., electronic medical records and computer-assisted coders systems [5]. Automated charge-capturing processes and centralized information can streamline billing, reduce rates of human error, enhance revenue, and improve patients’ payment [6,7].

The Ministry of Health in Saudi Arabia primarily provides healthcare services, covering about 80% of the governmental health services offered. Other government ministries provide healthcare services to their employees and dependents, such as teaching and military healthcare services. The Saudi Health system is under a major transformation to improve the efficiency and quality of care services. According to the Saudi 2030 vision, the country will have a comprehensive, practical, and integrated healthcare system [8]. The system is to be restructured, approaching value-based healthcare in which financial sustainability and preventive care are introduced and set at the core of the new system. A national intention is to address this by emphasizing privatization in their Vision 2030 statement [9]. Meanwhile, the healthcare system has suffered from high demand, escalated costs, accessibility and equality concerns, and poor coordination and continuity of care [10]. Similarly, another study unveiled escalated costs and unequal accessibility during this transition [9]. Saudi Arabia plans to invest USD 65 billion in healthcare infrastructure for national transformation and Vision 2030. By 2030, the government aims to increase the private sector’s contribution to the healthcare sector from 40% to 65% while privatizing 290 hospitals and 2300 primary health centers [11]. The Health Sector Transformation program recognizes the importance of privatization in achieving its objectives. The program seeks to improve and expand primary care facilities nationwide, establish new medical cities, and modernize laboratory and radiology services through strategic partnerships with private sector organizations. The Saudi Arabian government-funded healthcare system is planning to move away from the current budgeting model known as the global budget model. This model involves making a lump-sum payment for a specified period of health services. Instead, policymakers should prioritize financial budgeting by transitioning towards funding models based on activity and outcomes. This shift will help to ensure better financial management and more efficient use of resources in the healthcare system. Revenue cycle management (RCM) is a process that involves using technology to capture all streams of revenue generated from healthcare service provision. This study sheds light on the topic by hypothesizing that implementing RCM in governmental hospitals will be straightforward. Furthermore, there is no significant difference in the adoption of RCM between governmental and for-profit hospitals.

Literature Review

Since healthcare services began being provided for fees, revenue cycle management (RCM) has played a crucial role in the healthcare industry. RCM has streamlined the billing process in private healthcare in the US, particularly by implementing the comprehensive coding system (ICD-10 AM, ICD-10 PCS) and resulting in more efficient revenue collection [12]. In contrast, less emphasis is placed on RCM in countries running free national healthcare systems, like the United Kingdom [13]. Countries with growing private sector participation, like Saudi Arabia, can significantly benefit from RCM to streamline the private sector payments and justify the expense and budget allocation. Governmental hospitals differ from not-for-profit organizations (NPO), as they have different financial needs, and recommendations like revenue stream diversification are seldom possible [14]. In the United States, RCM has maximized profit margins, enhanced cash flow and equity capital, and improved financial viability by facilitating equity growth [2]. Hospitals with public-insured patients have experienced more significant revenue generation through RCM systems than those with private-insured patients [15]. Experiences from other nations reveal positive impacts upon introducing RCM in streamlining revenue sources, like Indonesia, India, and Kenya [16,17,18]. While in the Saudi context, few studies have assessed the impact of compiling data from clinical management systems, like bed management systems or patient scheduling, into RCM systems and have resulted in increased revenues [6].

Establishing an effective RCM system in Saudi governmental hospitals for healthcare reimbursement is paramount in capturing activities, driving outcomes, justifying budget allocation, and generating revenue. As the Ministry of Health (MOH) moves forward with regulating corporatized payers and providers, it is imperative to exercise caution and attention to detail when implementing RCM functions. Precise coding and documentation of clinical activities are crucial for accurate recording, efficient RCM, and revenue generation for governmental healthcare organizations. Therefore, it is important to assess the readiness of governmental hospitals to adopt an activity and the outcomes budgeting model. A comprehensive analysis of the status of RCM in Saudi hospitals is yet to be conducted. It is, therefore, essential to gain relevant knowledge to effectively identify potential obstacles that could impede a seamless transition toward activity-based budgeting. Furthermore, there is no study assessing the status of RCM in Saudi hospitals and knowledge is needed to shed light on this major transition with identifying barriers that can hinder smooth journey toward activity-based budgeting. The primary objective of this study is to examine the feasibility of implementing revenue cycle management (RCM) in Saudi Arabia’s governmental hospitals. This entails a thorough investigation of the current financial management and RCM principles from the perspective of key stakeholders. Furthermore, the study aims to offer practical recommendations on how RCM can be implemented and effectively utilized.

## 2. Materials and Methods

This qualitative study in which an interview approach was used to collect data to address the purpose of the study. Participants were sampled from governmental hospitals and recruited on a purpose basis. The study participants were the key informants who assumed executive positions in hospital financial management. The study’s main objective was to cover the major tertiary hospitals in Riyadh, Saudi Arabia. Within the region, there are approximately 11 tertiary hospitals, six of which are overseen by three distinct organizations. To ensure representation of each organization, one hospital from each was chosen. The study excluded a hospital that specializes exclusively in eye treatments. The research focused on key informants from a targeted sample of seven hospitals. The interviews aimed to solicit information and assess the feasibility of applying RCM through a deeper understanding of the constraints and opportunities that resulted during the implementation of RCM. Semi-structured interviews were conducted and analyzed using thematic analysis. Information about sensitive issues and statistics, like hospital expenses, budget, and services provided, were avoided. Instead, the interview focused on the key informants’ perspectives and anticipated challenges facing governmental hospitals regarding financial management. The interview was commenced by eliciting the key informants’ comprehension of RCM. Subsequently, inquiry was made about their perspective on the existing principles employed in hospital management and whether they believed these strategies promoted patient care and enhanced hospital operations. After that, informants were asked about their familiarity with utilizing RCM in hospital management and any obstacles that they encountered. Lastly, they were requested for their suggestions on how to tackle any challenges mentioned. The interviews were recorded and transcribed later, after which the data was gathered and organized. Consensus was achieved through the involvement of a third investigator in cases of disagreement between the two investigators. Ethical approval was obtained from King Abdullah International Medical Research Center (KAIMRC). Consent forms were collected before each interview, including the study’s purpose, and ensuring confidentiality on how data will be managed and disclosed.

## 3. Results

A semi-structured interview was employed to collect in-depth information and to follow up on any prompts or ask clarifying questions. Key informants from seven governmental hospitals were interviewed, and to protect their identity and maintain confidentiality, the interviews were coded as INT1, INT2, INT3, and so forth to protect the identity of the key informants. On average, each interview lasted 75 min and spanned from 50 to 120 min. These interviews were recorded and transcribed.

The initial interview question was related to respondents’ understanding of RCM. Answers varied in detail, but all understood the general principle. In response to the second question about the perspective of key informants on the existing principles utilized in hospital financial management, the respondents highlighted various external and internal challenges. They mentioned that the increasing demand for services, limited resources, and associated costs are significant external challenges that hospitals face. On the other hand, internal challenges are related to organizational factors. Despite the importance of accountability in government hospitals, several obstacles make it challenging to achieve this goal. For instance, bureaucratic rigidity and the high cost of governance are significant challenges. The respondents emphasized that accountability required reporting on how well an organization fulfilled its mission and achieved financial sustainability. However, government hospitals do not prioritize this aspect. As a result, there is a need for an internal audit role to establish a monitoring system that ensures organizational resources are used efficiently and effectively. The internal control system comprises five key components: control environment, risk assessment, control activities, information and communication, and monitoring. Among the seven surveyed hospitals, two reported operating business clinics that utilize a fee-for-service model to generate revenue. They determine charges by referencing the charge description master document and record expenses accordingly. However, the hospitals’ billing systems are not integrated with their information systems and function independently. Table 1 illustrates the main questions of the study and the themes of the key informants’ answers.

During an inquiry about integrated RCM, the interviewees were asked about their current systems in the hospitals. The respondents identified different levels of integration within their organizations and acknowledged that the process of RC, including highly integrated RC, was ongoing. INT6 mentioned they were still building their health economics department and assigning staff. INT7 confirmed the implementation of the RCM system, but it has not yet been utilized. INT1 stated there have been no considerable advancements in the implementation journey and said, ‘We have not seen many advancements when it comes to how our priorities are implemented’.

For this portion of questions, we noticed inconsistency and scope creep in how respondents envision the RCM; no single hospital has fully implemented the RCM system.

The following questions identify challenges respondents face when reforming or creating integrated RC systems and processes in their organizations. The challenges have been grouped into themes below. Implementing the RCM system lacks a clear vision due to competing interests. INT1 aims to create a vision that benefits the organization and satisfies all parties. INT1 noticed that the prevalent mindset is often competitive, focusing on winning rather than collaboration. INT3 mentioned conflicting interests due to varying approaches, such as casual clinical practice, professional disciplines, and high-performance culture. According to INT6, physicians at their institution are unclear about their role in implementing RC and its benefits. The staff did not prioritize saving money or reducing the financial burden for the institution or government, leading to conflicting interests and inconsistencies in the purpose and reality of the RCM system.

The challenge for governmental hospitals in implementing RCM is ensuring accountability for integrating it with existing clinical and administrative systems. Furthermore, proper policies and procedures are not yet proposed or implemented. INT5 recommended providing staff with orientation, training, and manuals to ensure accountability and prevent errors. INT3 reported that clinical coding and clinical documentation improvement courses for healthcare providers confirmed consistent and accurate coding across the organization. Other interviewees expressed their conviction in the benefits of consistent and holistic training across the organization but mentioned it was not being implemented.

The staff’s resistance is a second theme in the context of the RCM’s challenges. INT7 explained that their organization had the necessary understanding, systems, and processes but lacked the human resources and expertise in specific areas and project management aspects. Several interviewees echoed the need for a change in mindset among staff members, including motivation to adhere to provider principles, effective integrated RC, existing processes, and improvements. INT1 succinctly encapsulated this by stating that ‘this mindset needs to change here’. INT3 identifies resistance to change as a significant challenge in their organization. INT2, INT4, and INT7 noted that developing a perspective that revenue is their core business may impact governmental hospitals’ clinical practice, service documentation, and financial sustainability optimization. They describe the current situation as the status quo and emphasize the need to shift this mindset for improved effectiveness and efficiency in the future.

According to the respondents, the RCM system’s successful implementation is hindered significantly by the need for process redesign and proper system functionalities. INT4 stressed the importance of integrating and connecting payers, insurance companies, and healthcare providers to reduce incompatibilities and improve systemic efficacy. INT3 notes that the current system structure, general ledger, cost centers, revenue attribution, pricing, and information flow do not support the revenue cycle. INT1 expresses concerns about siloed systems hindering standardization and resolving systemic barriers to establish a centralized system that supports the RCM processes.

All respondents agree on the importance of exercising project management to lead RCM implementation and digital transformation. INT2 pointed out that significant transformational processes, like implementing integrated RC, often suffer from haste. INT1 confirms the progress made but notes the need to address improper management of the RCM project. INT3 similarly explains that systems might miss out on revenue cycle or standard of care standards for physician compensation. These challenges are prominent in rural areas, which are shared globally. INT4 suggests that providers should implement their RCM based on their system, process, and people while acknowledging the differences in landscapes. Providers should train their employees, facilitate change management, create a transformation plan, and follow it. There is no one-size-fits-all solution. The healthcare provider can implement a system to maintain experts within their created contexts. INT4 agrees and emphasizes effective monitoring and evaluation processes to ensure fit-for-purpose systems and procedures. INT6 emphasizes communication importance. INT1 emphasizes patients’ perspective, highlighting dissatisfaction with claims’ processing time. According to INT1, bringing in stakeholders can create direct and holistic patient benefits. INT1 explains that patients are more likely to continue using a medical facility if they receive prompt and honest service, even if they receive a rejection. This finding integrates the financial and administrative side of the provider’s operation with the patient’s experience, including medical treatment perception and the physician’s perspective. According to INT6, healthcare providers should involve physicians more directly to ease the transition for institutions and patients. The physicians serve as middlemen, building internal capacity, while seeking crucial external support.

Some respondents (INT2, 3, 5, 6, and 7) emphasized the importance of external expertise and proper vendor management. While there were varying opinions on the extent to which external expertise should be sought, the value of accessing some external capacity was widely acknowledged. INT7 explains that building a revenue cycle management system requires a cohesive approach rather than just patching together existing pieces. If you are unfamiliar with the revenue cycle, it is best to seek help from experts who can build the infrastructure and guide you in starting strong. The preferred approach from INT7’s perspective is to utilize expertise during the first implementation phase to ensure effective and aligned systems.

## 4. Discussion

The healthcare industry faces several hurdles, including escalating healthcare expenses, concerns over operation efficiency, and a shortage of healthcare professionals [14]. The efficient management of the healthcare revenue cycle is of utmost importance for healthcare facilities to effectively handle the administrative and clinical functions associated with claims processing, payment, and revenue generation. This process involves identifying, managing, and collecting patient service revenue and is a crucial aspect of healthcare administration. The hospital billing process involves several essential steps that must be followed for success. These steps include preregistration, registration, charge capturing, claim submission, remittance processing, insurance follow-up, and patient collections. By following these steps, healthcare facilities can ensure that the billing process is streamlined and efficient, leading to increased revenue generation. In addition, the hospital expenditure cycle comprises two types of expenses: capital and revenue. Capital expenditures are large, one-time purchases of fixed assets that generate revenue over an extended period, while revenue expenditures are ongoing operating expenses that cover short-term expenses necessary for daily hospital operations. The government pays for 75% of all healthcare expenditures. However, the operating expenditure of governmental hospitals can range from tens of millions to hundreds of millions of dollars, depending on their size. Proper management of both types of expenses is vital for the financial stability of healthcare organizations and requires careful planning and execution.

The research aimed to survey the key stakeholders regarding their perspectives on financial management and the RCM systems in Saudi governmental hospitals. Specifically, the primary phase gathered data to understand current financial practices and RCM knowledge and provide recommendations for RCM implementations.

Managing finances and developing effective strategies in government hospitals has been challenging and has caused conflict. Nonprofit hospital systems in the United States sought non-operating income but also had to deal with cost inflation and unpredictable government reimbursement policies [19]. Furthermore, the government allowed charitable organizations to establish tax exemption subsidiaries with profit-making goals. They were also engaged in mergers and acquisitions, some of which were anti-competitive.

Government hospitals often face internal challenges that hinder accountability due to organizational factors. Despite the importance of addressing this issue, bureaucratic rigidity and high governance costs make it challenging. Respondents have highlighted the importance of hospitals reporting on their financial sustainability and mission fulfillment. Regrettably, these aspects are not prioritized in government hospitals. Therefore, it is crucial to establish an internal audit role to monitor the efficient and effective use of organizational resources. The literature suggests that a robust financial reporting system reduces errors and mismanagement and improves financial performance. Kewo posited that enhancing financial accountability can lead to improved financial performance [20]. Likewise, Wynn-Williams demonstrated that public sector organizations can strengthen their financial performance by implementing enhanced accountability reporting systems, incorporating internal and process benchmarking, and increasing public documentation [21]. These findings suggest that effective accountability mechanisms play a crucial role in enhancing the financial performance of organizations. According to Appelbaum and Batt (2021), healthcare decisions may be influenced by for-profit motives, blurring the line between nonprofit and for-profit organizations [19]. However, the literature also provides evidence for the effectiveness of RCM, especially with the constant advancements in technology [7,22,23]. In this research, physicians and administrators had conflicting aims and objectives. This confirms Appelbaum and Batt’s (2021) study on cultural conflicts arising from changes in healthcare organizations’ financial systems and purposes, including governmental hospitals [19]. According to Turnbull et al., (2018), the multiplicity of interests among staff in healthcare settings, especially in governmental hospitals, can lead to tensions among different groups [24]. Turnbull et al. (2018) argue that boards of directors and hospital managers may have interests that differ from those of clinicians and patients [24]. Hospital administrators are responsible for maintaining financial stability and expanding the institution’s goals, including providing healthcare to the community, as well as patient care, teaching, and research. In some cases, administrators may feel pressure to prioritize the interests of the federal healthcare system, regulators, political figures, local employers, insurers, or hospital board members over those of patients, which can lead to decisions that are not in the best interest of patients [24].

This theme focuses on the challenges of implementing RCM in Saudi Arabian governmental hospitals, specifically related to staff capabilities and know-how in the face of systemic changes. This aligns with Foroughi et al.’s (2022) systematic review of hospitals during economic challenges [25]. According to Foroughi et al., (2022), short-term policies in 36 countries hindered hospitals from contributing to universal healthcare, ultimately impacting the quality and resilience of hospitals [25]. Staff would have resistance to implementing measures to improve patient care and, at the same time, maintain financial stability.

According to the respondents, inconsistencies and potential service gaps were caused by staff shortage of knowledge and skills, particularly in RCM. This aligns with Aspirion’s (2020) recommendation for staff technical training on complex RCM and patient access [26]. Aspirin (2020) emphasizes the importance of staff making informed decisions, asking appropriate questions, and obtaining accurate information [26]. Being skilled in health system technologies and procedures is not enough, as staff must also be able to handle complex claims and improve patient satisfaction through training. This training can also reduce internal errors. Gorke (2016) found that despite the US’s established financial system, some institutions underinvest or ignore the importance of RCM and its supporting parts [27]. Healthcare margins are lower than other service-oriented industries and are heavily regulated. Gorke (2016) notes that accurate billing and revenue management are crucial for healthcare businesses, including governmental hospitals [25]. This requires staff training and accountability systems and adapting to changing global healthcare management roles [25].

The study participants found inconsistencies in billing and revenue processes within their organizations, even across departments, leading to potential revenue loss and inefficiencies. According to Optum (2022), there is a substantial unpaid provider reimbursement amounting to millions of dollars every year. The average facility experiences a loss of 2% to 3% of its yearly revenue due to preventable leaks [28]. Providers face a significant problem due to underpayments, improper denials, and other reimbursement anomalies that cause a slow and constant income loss or leakage, often going unnoticed and seeming inconsequential [28]. Therefore, hospitals need to standardize their processes and attain accurate data on revenue performance to avoid needless financial losses due to correction of payment variations. However, the disparities in revenue collection systems reflect different political ambitions and priorities in different countries, despite a common need for effectiveness and efficiency, and are potentially far-reaching and cumulative.

According to Appelbaum and Batt (2021), healthcare has become financialized due to two parallel processes. Nonprofit hospitals increasingly turn to non-healthcare-related financial strategies to survive, while financial actors have entered the industry because they view it as a lucrative investment [19]. Emphasizing the advantages of proper RCM systems and processes is crucial. The results of this study indicate that significant reform is necessary to pursue efficiency and effectiveness goals in public hospitals. According to Al Asmri et al. (2020), the Saudi healthcare system is undergoing a transition. It requires improvement due to increasing demand, high service costs, unequal access, a malfunctioning eHealth system, poorly coordinated interdepartmental interactions, and an overly centralized administrative structure [17]. This study confirms research participants’ concerns about rising costs and inequality in healthcare provision. While the National Vision 2030 statement emphasizes privatization to address these issues, standardization and increased revenues for governmental hospitals are necessary for an effective transition [16].

According to the research participants, external expertise is necessary due to the lack of consistent capabilities across Saudi governmental hospitals. However, caution was expressed regarding the degree of involvement, partnership, and relationship with vendors. Adaptability is crucial for survival and seizing opportunities in supply chain disruptions and offline purchasing channels. Adaptability may refer to how businesses interact with partners and agencies in the short term [29]. However, in the long run, it will require new alliances and unconventional partnerships, such as strategic mergers and acquisitions. A study by Al-Hanawi and Qattan examined Saudi Arabia’s healthcare system, focusing on the challenges of medical professionals and ministry representatives [29]. They investigate the feasibility of integrating market values with public services by analyzing the experience of healthcare modernization in the United Kingdom. Al-Hanawi and Qattan’s study has clarified that the current government funding model supporting the Saudi healthcare system is unsustainable [30]. The research participants in this study have further corroborated this, and urgent action is required to restructure the system. Alternate management and funding methods should be allowed into the public healthcare system, drawing on external expertise and capabilities. Public–private partnerships are an effective way to finance Saudi Arabia’s healthcare system and improve health service quality. Research of Al-Hanawi and others confirms that collaborating with external partners in RCM and other healthcare areas in Saudi Arabia is undeniably challenging, a sentiment strongly echoed by participants [30].

This study aimed to delve into the various viewpoints of key stakeholders regarding the current financial management and RCM principles. After carefully examining the transcripts of the interviews, we have encountered six prominent themes that have consistently emerged across all the interviews. These themes serve as crucial evidence, showcasing increasing understanding and knowledge regarding RCM in governmental hospitals in Saudi Arabia.

While the advantages of adopting RCM were acknowledged by those involved, the importance of receiving sufficient support from upper management was strongly emphasized. To navigate this substantial transformation, it is necessary to establish a clear vision for the desired outcome, allocate crucial project management resources, and involve clinical and administrative stakeholders early. It has been widely acknowledged that there is a significant gap in terms of skills and resources among the staff. To tackle this issue, recommendations have been put forth to implement concrete steps such as providing comprehensive staff training programs, ensuring accountability, and fostering continuous collaboration with external experts and organizations. These measures aim to enhance the workforce’s capabilities and improve overall organizational effectiveness.

It is important to note that the study in question is exploratory. In addition, the data was gathered via interviews, which could be influenced by self-reporting and presentation of biases. While the recommendations put forth by the study may have relevance for governmental hospitals in Saudi Arabia, it is essential to exercise caution when attempting to generalize these findings. This is because there may be significant differences in terms of organizational size, maturity, administration, and finances across various healthcare facilities. Furthermore, the study revealed that certain respondents harbored apprehensions concerning their non-governmental revenue streams, given that they operated small business clinics.

Due to the lack of studies in the literature, it is highly recommended that future studies thoroughly evaluate the shift from hospitals that the government funds to those that are self-operated. Such studies should cover a wide range of aspects, including alterations in the hospitals’ vision, mission, structure, policies, and procedures, and the establishment of appropriate practices. This in-depth analysis will provide a more comprehensive understanding of the challenges and opportunities during this transition period.

## 5. Conclusions

The study investigated how governmental hospitals in Saudi Arabia implement financial management and RCM principles. Six themes emerged from the interview analysis, indicating changing attitudes towards RCM. Despite variations, participants acknowledged its benefits but emphasized the need for a clear vision and cultural change, including accountability and resource allocation, to ensure successful adoption and financial sustainability. The study also offers practical recommendations for effective and equitable RCM implementation, such as staff training, external partner involvement, and consistency. The healthcare system in Saudi Arabia requires new approaches to address current gaps in RCM and revenue optimization. Recognizing challenges and implementing solutions for RCM in government hospitals is crucial. Future studies must comprehensively evaluate the transition from government-funded to self-operated hospitals, including changes to vision, structure, policies, procedures, and best practices implementation. This will provide a better understanding of challenges and opportunities during the transition.

## Figures and Tables

**Table 1:** Main study inquiries and responses to various themes.

Inquires	Answers and Themes
The key informants’ comprehension of RCM.	There is an agreement on how the key informants understand RCM. Below are some answers. ▪ RCM is a software-enabled process that aims to capture all bills within a single patient care episode. ▪ RCM is the process of generating bills based on the provided services during the care process. ▪ The process of RCM involves recognizing, overseeing, and gathering revenue for the services provided to patients. It is a crucial aspect of healthcare administration that ensures the effective management of patient service revenue. ▪ A well-designed RCM system improves billing and collection cycles by accurately processing payments. ▪ RCM incorporates patient demographic and administrative data into care.
Current hospital financial management	Challenges that exist. - External include increase demand, limited resources, and associated costs. - Organizational factors include accountability and Internal Control System within government hospitals. - Small scale business clinics
Obstacles encountered during the implementation of the RCM system	As of now, no hospital has fully implemented the RCM system. Obstacles include the following: - The RCM system lacks a clear vision. - Accountability and proper policies and procedures. - The staff’s resistance. - The need for process redesign and proper system functionalities. - The importance of exercising project management.
Recommendations to tackle any challenges mentioned for proper RCM implementation	- Significant transformational processes, including vision, governance, and accountability. - Training. - Effective monitoring and evaluation processes. - Communication must also be emphasized. - Bringing patient perspective into focus. - Bringing in all stakeholders can create direct and holistic benefits for patients.

## Data Availability

Not applicable.
